# Clinical value and potential association of Rab1A and FoxM1 aberrant expression in colorectal cancer

**DOI:** 10.1038/s41598-020-77182-z

**Published:** 2020-11-19

**Authors:** Menglin Xu, Xinyu Shao, Haoran Li, Zhengrong Zhang, Chunli Zhou, Zhengwu Cheng

**Affiliations:** 1 Department of Oncology, The First Affiliated Hospital of Wannan Medical College, Wuhu, 241000 China; 2 Department of Gastroenterology, The Affiliated Suzhou Hospital of Nanjing Medical University, No.242 Guangji Road, Suzhou, 215006 Jiangsu Province China; 3 Department of Gastrointestinal Surgery, The First Affiliated Hospital of Wannan Medical College, No. 2 Zheshan West Road, Jinghu District, Wuhu, 241000 Anhui Province China

**Keywords:** Oncogenes, Prognostic markers

## Abstract

Colorectal carcinoma (CRC) is one of the most common malignancies with a dismal 5-year survival rate. Our recent study indicated that Rab1A expression was closely related to GLI1 expression. A previous study shows that aberrant overexpression of GLI1 promotes colorectal cancer metastasis via FoxM1 overexpression. However, the potential correlation between Rab1A and FoxM1 in CRC remains elusive. Immunohistochemistry was performed to investigate the association of the expression of Rab1A and FoxM1 and to determine the prognosis in 135 CRC tissue and adjacent normal tissues. Using Oncomine datasets, we found that Rab1A and FoxM1 mRNA were obviously upregulated in CRC tissues compared to normal tissues. Additionally, the expression of Rab1A and FoxM1 was significantly higher in CRC tissues than that in normal tissues. Rab1A expression was positively correlated with FoxM1 expression in CRC, especially in TNM stage III. In addition, Rab1A and FoxM1 overexpression was found to be significantly correlated with poor prognosis in CRC patients. Besides, both high expression of Rab1A and FoxM1 led to a worse prognosis than anyone low group, and both low expression of Rab1A and FoxM1 had a better prognosis than the anyone low group. Therefore, Rab1A and FoxM1 play crucial roles and could be used as clinical biomarkers in CRC.

## Introduction

Colorectal cancer (CRC) is a common malignant tumor of the digestive tract, which is associated with high morbidity and mortality worldwide^1,2^. Compared to that in other developed countries, CRC has a high incidence in China^3^. Recently, comprehensive treatment modalities, such as resection and adjuvant chemotherapy, have been widely used. However, the long-term prognosis in patients remains relatively unsatisfactory mainly due to metastasis and resistance to chemotherapy^4,5^. Thus, it is necessary to identify novel targets to improve treatment outcomes in patients with CRC.

Rab proteins have been identified as housekeeping proteins that regulate intracellular membrane dynamics^6^. As a member of the RAB family, Rab1A has been verified to mediate vesicular trafficking^7^. Recent studies reveal that Rab1A is involved in regulating signal transduction^8^, cell autophagy^9^. Previous studies indicated Rab1A acted as an oncogene and Rab1A overexpression promoted a worse prognosis in multiple cancer types such as gastric cancer^10^ and hepatocellular carcinoma^11^. In addition, Rab1A overexpression has been positively associated with proliferation, migration, and drug-resistance in various cancers^12,13^.

Forkhead box M1 (FoxM1) belongs to a large family of Forkhead transcription factors^14^. Recent studies indicate that FoxM1 plays a significant role in cell-cycle progression, where endogenous FoxM1 expression peaks at the S and G2/M phases^15^. In addition, FoxM1 overexpression has been linked with cancer progression and poor prognosis in several human cancers, suggesting that FoxM1 might play a crucial role in tumor initiation^16,17^.

A study shows that aberrant overexpression of GLI1 promotes the proliferation and migration of colon cancer cells through the transcriptional activation of FoxM1^18^. Nowadays, our research revealed Rab1A expression was closely related with GLI1 expression in gastric cancer^19^. However, the potential correlation of Rab1A and FoxM1 in both expression and prognosis remains largely unknown in CRC. The aim of this study was to explore the clinicopathological features and the potential associations between Rab1A and FoxM1 and to determine how they can be used as novel targets in the individualized treatment of CRC.

## Results

### Expression levels of Rab1A and FoxM1 in gastrointestinal cancers

**Figure 1:**
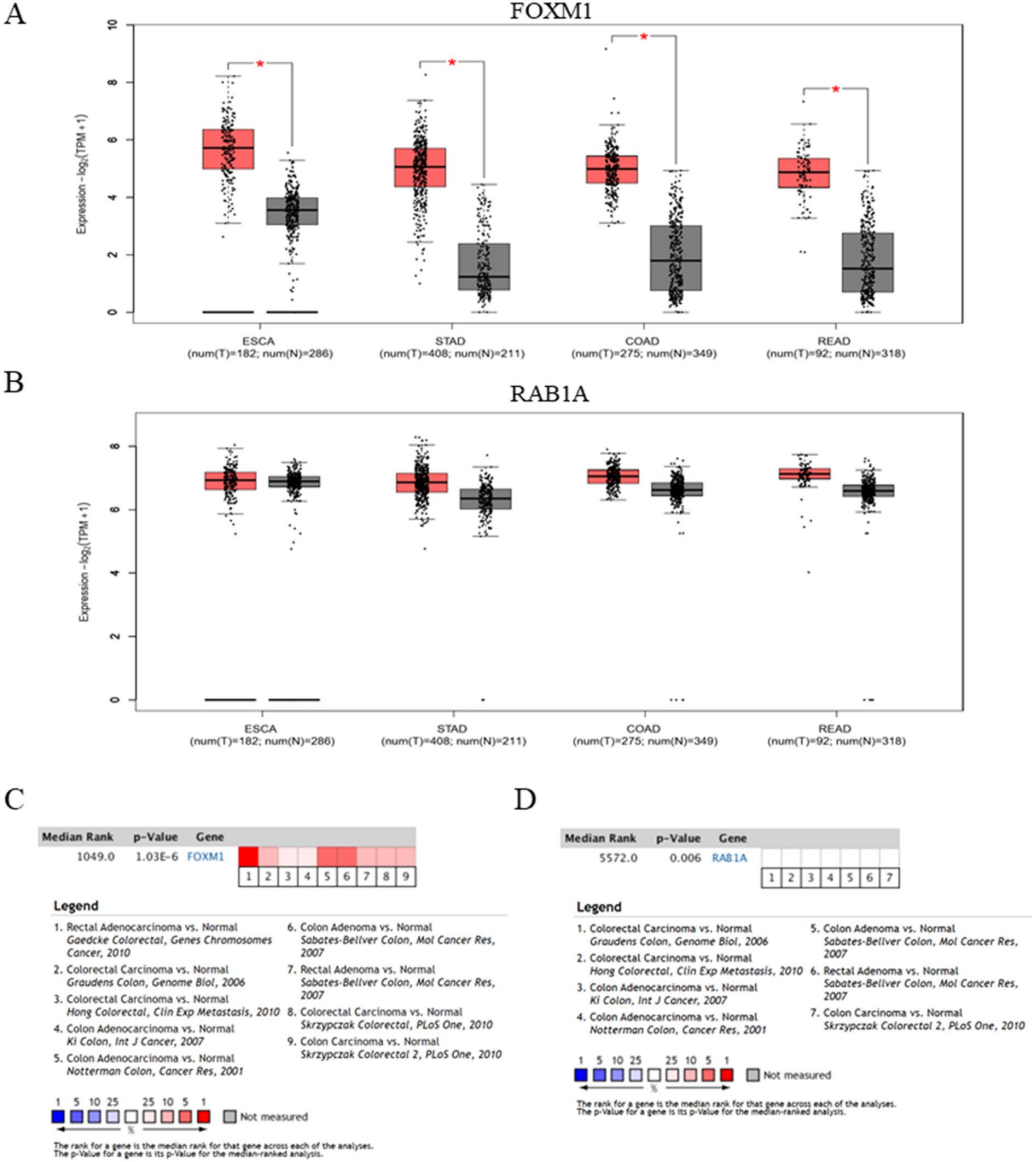
Expression of Rab1A and FoxM1 in gastrointestinal cancers in the GEPIA Platform and Oncomine (https://www.oncomine.org). (**A**) Comparison of FoxM1 levels in gastrointestinal tumor and paired normal tissues. (**B**) Comparison of Rab1A levels in gastrointestinal tumor and paired normal tissues. (**C**) Comparison of FoxM1 mRNA expression in CRC and normal tissues across 9 Oncomine datasets. (**D**) Comparison of Rab1A mRNA expression in CRC and normal tissues across 7 Oncomine datasets. * P < 0.05.

We determined the expression of Rab1A and FoxM1 in gastrointestinal cancers via the GEPIA Platform. These findings suggested that FoxM1 was significantly overexpressed in esophageal carcinoma (ESCA), stomach adenocarcinoma (STAD), colon adenocarcinoma (COAD), and rectal adenocarcinoma (READ) tumor tissues compared to paired normal tissues (*P* < 0.05) (Fig. 1A). Rab1A expression was moderately higher in ESCA, STAD, COAD, and READ tumor tissues compared to normal tissues; however, this difference was not significant (*P* > 0.05) (Fig. 1B). Pooled analysis of CRC and normal tissues across 9 Oncomine datasets indicated significant upregulation of FoxM1 mRNA in CRC tissues (*P* < 0.001) (Fig. 1C). Additionally, using 7 Oncomine datasets, we determined that Rab1A mRNA was obviously upregulated in CRC tissues compared to that in normal tissues (*P* = 0.006) (Fig. 1D).

### Expression levels of Rab1A and FoxM1 in different TNM stages of CRC

**Figure 2:**
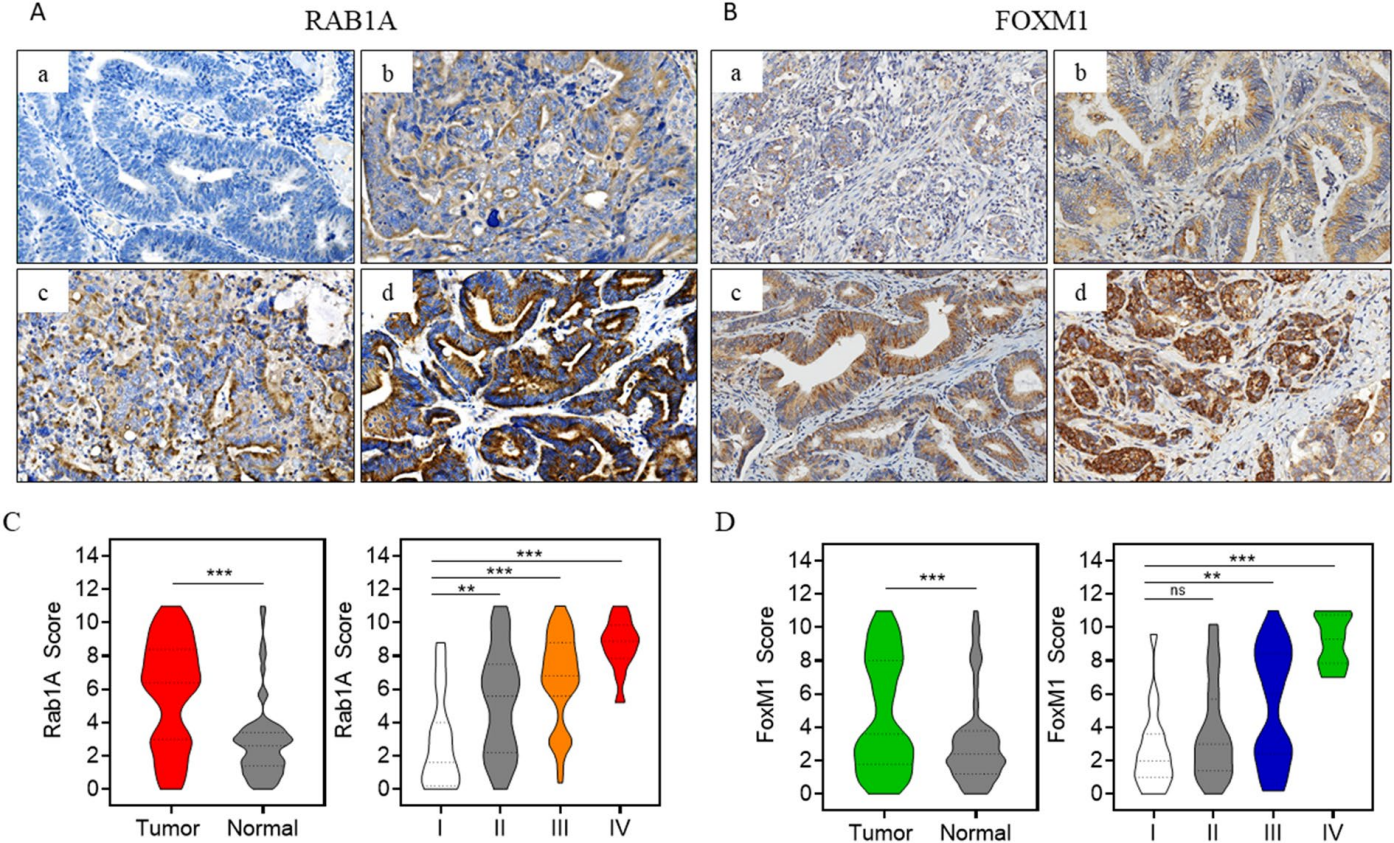
Expression of Rab1A and FoxM1 in 135 pairs of CRC and normal tissues. (**A**,**B**) Immunohistochemistry (IHC) staining of Rab1A and FoxM1 in CRC tissues (200 ×) (scale bar = 100 µm). The protein expression of Rab1A was negative (a), weak (b), positive (c), strong positive (d). (**C**) Staining scores after analysis of Rab1A expression in CRC tissues and adjacent normal tissues (left), and IHC analysis of Rab1A expression in CRC tissues in TNM I stage and TNM II, III, IV stages in patients (right). (**D**) Staining scores after analysis of FoxM1 expression in CRC tissues and adjacent normal tissues (left), and IHC analysis of FoxM1 expression in CRC tissues in TNM I stage and TNM II, III, IV stages in patients (right). ** P < 0.01, ***P < 0.001.

First, immunohistochemistry (IHC) staining was performed in 135 pairs of colorectal cancer and adjacent normal tissues to detect Rab1A and FoxM1 expression (Fig. 2A,B). We found that the expression of Rab1A and FoxM1 was significantly higher in colorectal cancer tissues than in normal tissues (*P* < 0.001, *P* < 0.001) (Fig. 2C,D). In addition, a subgroup analysis was performed to detect Rab1A and FoxM1 expression in different TNM stages. Our results revealed that Rab1A expression was remarkably higher in TNM stages II, III, and IV compared to that in TNM stage I (*P* < 0.01, *P* < 0.001, *P* < 0.001) (Fig. 2C). The levels of FoxM1 were significantly higher in TNM stages III and IV compared to that in TNM stage I (*P* < 0.01, *P* < 0.001) (Fig. 2D). There was no obvious difference in FoxM1 levels between TNM stages I and II (*P* > 0.05) (Fig. 2D).

### The association between Rab1A and FoxM1 expression and clinicopathological parameters in patients with CRC

We investigated the association between Rab1A and FoxM1 expression and clinicopathological parameters, and the outcomes are shown in Table 1. The results showed that the expression of Rab1A and FoxM1 was obviously linked with several clinicopathological variables, such as venous invasion (*P* = 0.029) and TNM staging (*P* < 0.001). However, no obvious differences existed between Rab1A and FoxM1 expression and the left clinicopathological variables (*P* > 0.05).

**Table 1:** Association between Rab1A and FoxM1 and clinicopathological factors in 135 patients with CRC.

	Rab1A			FOXM1		
	Negative	Positive	*P* value	Negative	Positive	*P* value
**Age (years)**						
≤ 60	19	38	0.855	28	29	0.151
> 60	28	50		48	30	
**Gender**						
Male	27	50	0.546	41	36	0.484
Female	20	38		35	23	
**Size (cm)**						
≤ 5	25	34	0.145	38	21	0.116
> 5	22	54		38	38	
**Depth of tumor invasion**						
T1–2	17	8	< 0.001***	21	4	0.002**
T3–4	30	80		55	55	
**Lymph node metastasis**						
Yes	34	32	< 0.001***	48	18	< 0.001***
No	13	56		28	41	
**Degree of differentiation**						
Well	42	69	0.156	64	47	0.506
Poor	5	19		12	12	
**Venous invasion**						
Negative	39	57	0.029*	60	36	0.035*
Positive	8	31		16	23	
**Neural invasion**						
Negative	36	55	0.123	56	35	0.096
Positive	11	33		20	24	
**TNM staging**						
I–II	34	30	< 0.001***	48	16	< 0.001***
III–IV	13	58		28	43	

*CRC* colorectal cancer.

*P < 0.05, **P < 0.01, ***P < 0.001.

### Rab1A expression is positively related to FoxM1 expression in CRC

**Figure 3:**
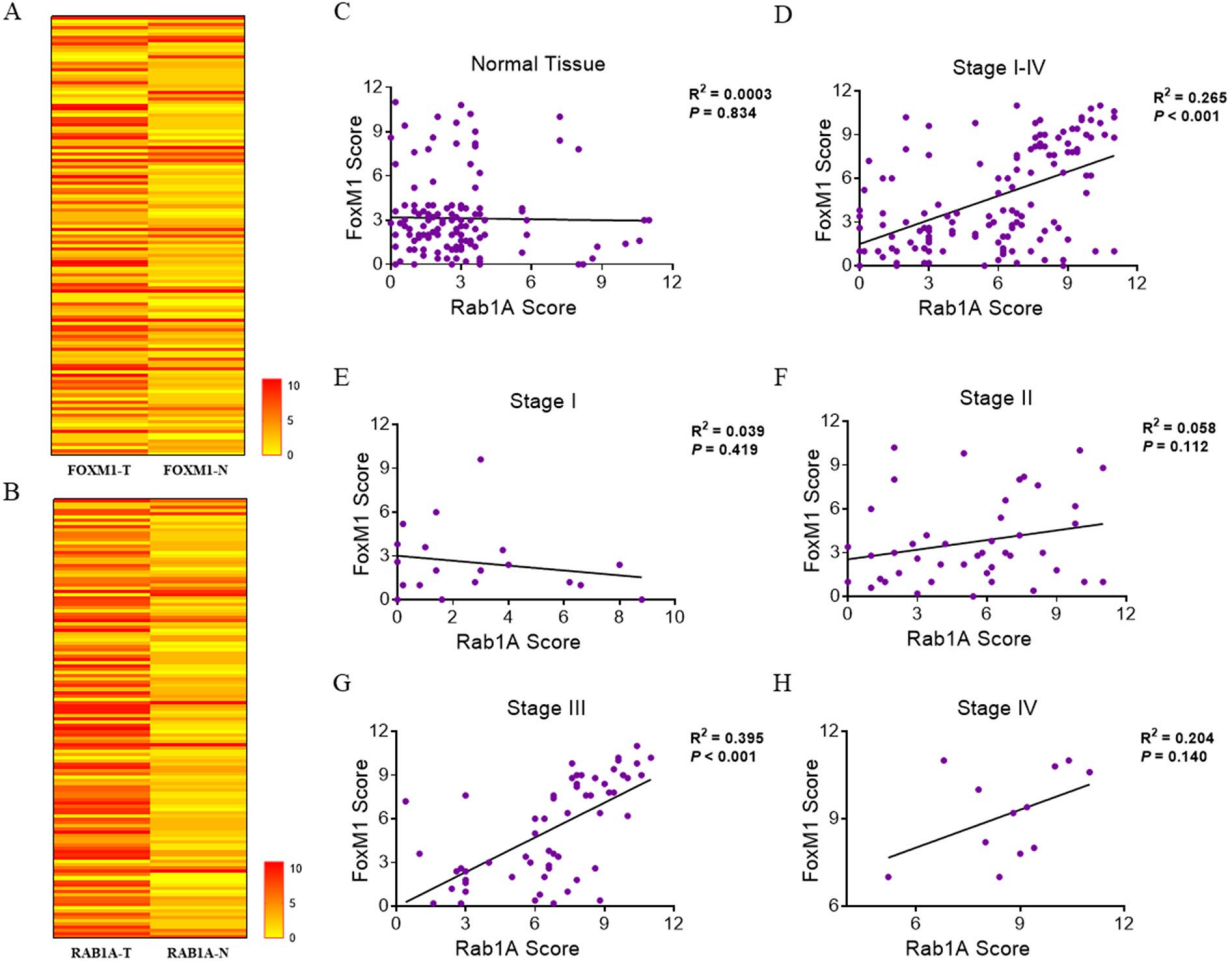
Analysis of the association between Rab1A and FoxM1 expression in 135 CRC tissues and paired normal tissues. (**A**,**B**) The association between Rab1A and FoxM1 expression in 135 CRC tissues and paired normal tissues is displayed as a heat map. Scatter plot analysis of the association between Rab1A and FoxM1 expression based on average IHC scores in normal tissues (**C**) and CRC tissues (**D**). Scatter plot analysis of the association between Rab1A and FoxM1 expression based on average IHC scores in TNM stage I (**E**), stage II (**F**), stage III (**G**), and stage IV (**H**).

**Figure 4:**
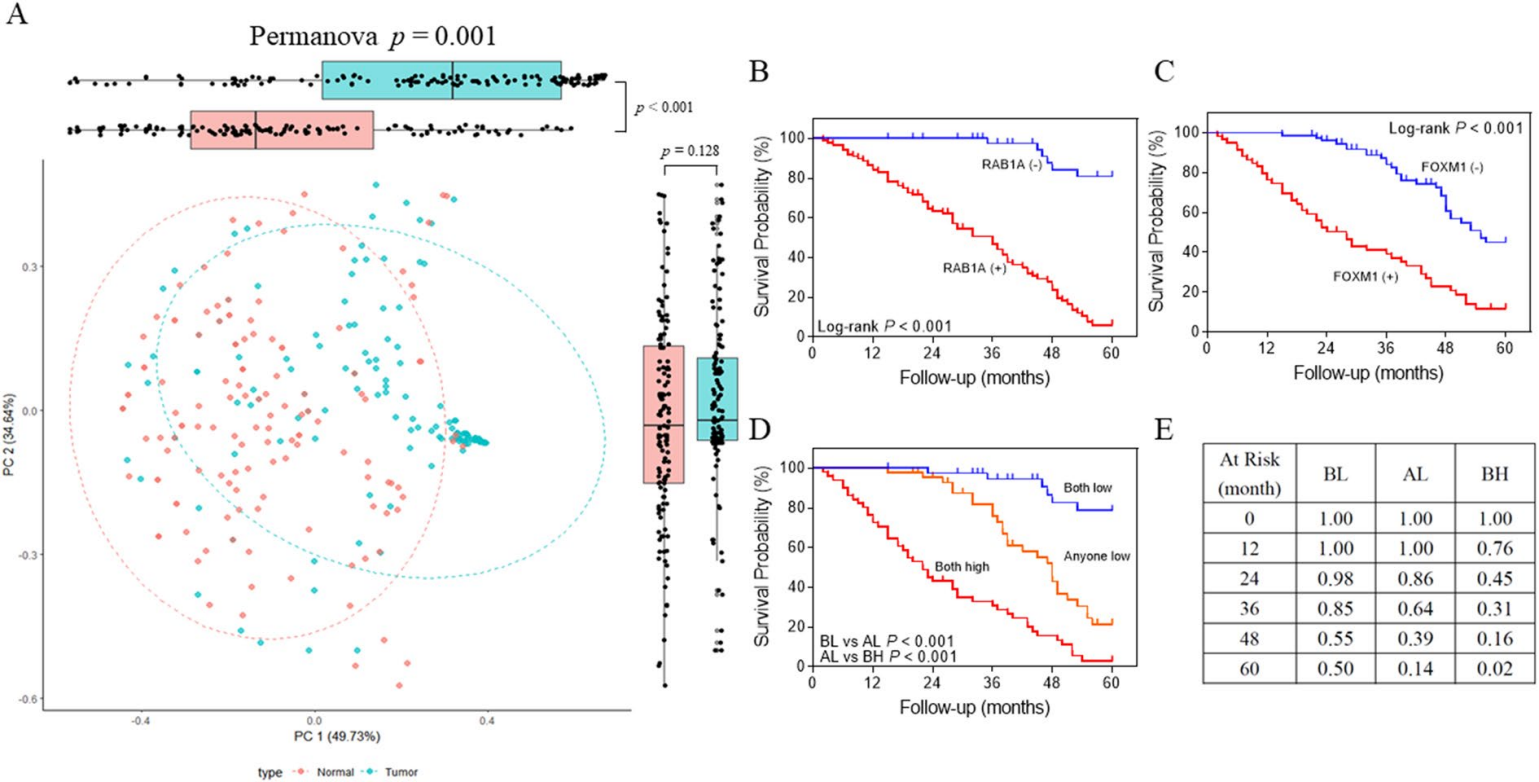
Association between Rab1A and FoxM1 expression and the influence of Rab1A and FoxM1 expression on prognosis. (**A**) Cluster analysis of 135 pairs of colorectal cancer (green) and normal tissues (red) based on Rab1A and FoxM1 IHC scores. The x- and y-axes represent the two most informative principal coordinates (PCs) of the PCoA; marginal boxplots describe the distribution of those values for the different groups. Color legends represent the respective variables under analysis. The paired Wilcoxon rank-sum test was used to compare the PC1 or PC2 values between groups; the p values were displayed beside the edge box. Kaplan–Meier survival analysis was used for the analysis of Rab1A (**B**) and FoxM1 (**C**) expression in 135 CRC patients. (**D**) Survival analysis of patients with both high (BH) in Rab1A and FoxM1 expression, any one low (AL) in Rab1A and FoxM1 expression, and both low (BL) in Rab1A and FoxM1 expression. (**E**) Annual survival rates among the three groups of BH, BL, and AL.

We first generated a heat map displaying Rab1A and FoxM1 expression detected in 135 CRC patients based on IHC analysis. Then, we compared the levels of Rab1A and FoxM1 detected in 135 CRC tissues and paired normal tissues in a scatter plot of IHC scores (Fig. 3A,B). The outcome suggested that no obvious association existed between Rab1A and FoxM1 expression in normal tissues (*P* = 0.834) (Fig. 3C). Interestingly, Rab1A expression was closely related to FoxM1 expression in tumor tissues (*P* < 0.001) (Fig. 3D), which was similar to the results in Table 2 (*P* < 0.001). Subgroup analysis of associations between Rab1A and FoxM1 expression was performed based on TNM staging. The outcome showed that Rab1A expression was positively associated with FoxM1 expression in TNM stage III (*P* < 0.001) (Fig. 3G), while no significant associations between Rab1A and FoxM1 expression were obtained in TNM stage I (*P* = 0.419) (Fig. 3E), stage II (*P* = 0.113) (Fig. 3F), and stage IV (*P* = 0.140) (Fig. 3H). According to the Rab1A and FOXM1 IHC score of human CRC and adjacent normal tissues, it was illustrated as a cluster analysis diagram to demonstrate the differences in expression of the two proteins in tumor and normal tissues (Fig. 4A). The tumor and normal samples were distributed in the PC1 and PC2 axes; the location of the dot was significantly different between tumor and normal tissues in the PC1 axis (*P* < 0.001) (Fig. 4A).

**Table 2:** Statistics of FOXM1 and Rab1A expression in CRC patients.

	Rab1A negative	Rab1A positive	*P* value
FOXM1 negative	39	37	< 0.001***
FOXM1 positive	8	51	

*CRC* colorectal cancer.

*P < 0.05.

### The influence of Rab1A and FoxM1 overexpression in the prognosis of patients with CRC

**Figure 5:**
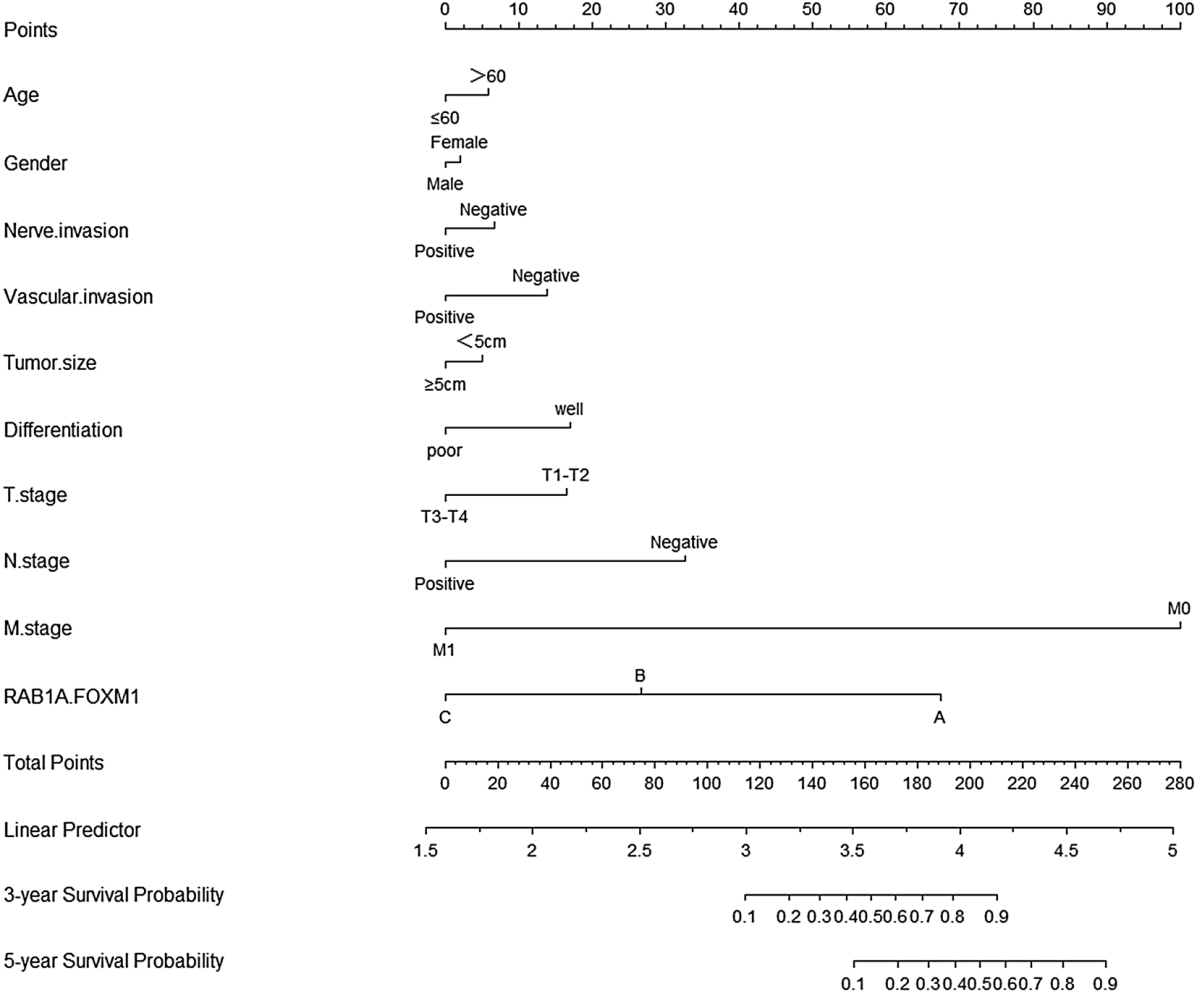
Nomograms to predict survival of CRC patients. Points of each variable were obtained via a vertical line between each variable and the point scale. The predicted survival rate was correlated with the total points by drawing a vertical line from the total points scale to the overall survival. A, B and C of Rab1A and FOXM1 line represent both low, anyone high, both high.

We first investigated the influence of Rab1A and FoxM1 overexpression on overall survival (OS) using Kaplan–Meier analysis in patients with CRC. Our findings indicated that patients with higher Rab1A and FoxM1 expression had shorter survival time compared to those with lower expression of Rab1A and FoxM1 in CRC (*P* < 0.001,* P* < 0.001) (Fig. 4B,C). In addition, both high (BH) expression group of Rab1A and FoxM1 led to a worse prognosis than any one low (AL) expression group (*P* < 0.001) (Fig. 4D). Conversely, both low (BL) expression group of Rab1A and FoxM1 had a better prognosis than any one low (AL) group (*P* < 0.001) (Fig. 4D). The annual survival rates among the three groups are shown in Fig. 4E. In addition, clinicopathological parameters and Rab1A and FoxM1 expression were used to estimate the 3- and 5-year OS (Fig. 5). The nomogram shows each prognostic factor as a score via a point scale and to get the total score (Fig. 5). Results revealed that the expression of Rab1A and FOXM1, which played a significant role in predicting the prognosis in patients with CRC, ranked second. The BL expression group had the best prognosis while the BH expression group had the worst 3-year and 5-year survival rates compared to the other two groups.

## Discussion

Rab1A is a small GTPase that regulates ER-to-Golgi vesicular transport^20^. Aberrant Rab1A expression has been linked to a range of human diseases such as Parkinson's disease and cardiomyopathy^21,22^. Previous studies report that Rab1A acts as an oncogene and is overexpressed in various types of cancers, including tongue squamous carcinoma^23^ and colorectal cancer^24^. In our study, we first explored Rab1A expression in CRC and paired normal tissues using 7 Oncomine datasets. Our results indicated that Rab1A mRNA was obviously upregulated in CRC tissues compared to the normal tissues. Besides, using IHC, we determined that Rab1A expression was significantly overexpressed in CRC tissues compared to normal tissues, which was consistent with the findings of a previous study^25^. Subgroup analysis showed that Rab1A expression was remarkably higher in TNM stages II, III, and IV compared to that in TNM stage I, which indicated that Rab1A expression might play a critical role in tumor initiation and development.

A study reports that Rab1A overexpression is positively related to clinicopathological parameters^19,26^. Our results indicated that Rab1A expression was obviously linked to the depth of tumor invasion, lymph node invasion, venous invasion, and TNM staging. Rab1A overexpression is positively correlated with poor prognosis in several gastrointestinal cancers, such as CRC^6^ and GC^13^. Consistent with these reports, our findings indicated that lower Rab1A expression indicated better prognosis in patients with CRC.

Our previous study indicates that Rab1A expression is closely related to GLI1 expression in gastric cancer ^19^. A recent study reported that GLI1 expression is closely associated with FoxM1 expression and that GLI1 expression elevation promotes colorectal cancer metastasis via FoxM1 overexpression^27^. Based on this perspective, the potential correlation of Rab1A and FoxM1 in expression and prognosis was explored in the current study. Pooled analysis of CRC and normal tissues across 9 Oncomine datasets revealed significant upregulation of FoxM1mRNA in the CRC tissues compared to that in normal tissues. In addition, using IHC analysis, the outcomes revealed that FoxM1 expression was significantly higher in CRC tissues than normal tissues, which was consistent with the results from previous studies^28^. In addition, we observed that FoxM1 expression was significantly higher in TNM stages III and IV compared to that in stage I, which was similar to the results of Rab1A expression level in vary TNM stage. Consistent with previous reports^29^, we found that increased expression levels of FoxM1 led to significantly worse overall survival in patients with CRC.

To investigate the potential correlation of Rab1A and FoxM1 in both expression and prognosis, first, we compared the levels of Rab1A and FoxM1 in a scatter plot of IHC scores. We found that Rab1A expression was closely related to FoxM1 expression in tumor tissues, whereas no obvious expression association in normal tissues was observed. Then, subgroup analysis of expression associations was performed based on different TNM stages. Results revealed that Rab1A expression was positively associated with FoxM1 expression in TNM stage III, indicating that Rab1A and FoxM1 might have promoted tumor progression. In addition, BH expression of Rab1A and FoxM1 led to a worse prognosis than AL expression, and BL expression had a better prognosis than AL expression. The above outcomes revealed that the overexpression of Rab1A and FoxM1 had a greater impact on OS than negative expression.

However, our study had some limitations. No in vitro or in vivo experiments were performed. To summarize, Rab1A and FoxM1 may be used together in predicting patient prognosis and may be potential novel targets for individualized therapy of CRC.

## Conclusion

We used Oncomine datasets and showed that Rab1A and FoxM1mRNA were obviously upregulated in CRC tissues compared to that in normal tissues. Additionally, the expression of Rab1A and FoxM1 was obviously higher in CRC than in normal tissues. Rab1A expression was closely associated with FoxM1 expression in CRC, especially in TNM stage III. In addition, Rab1A and FoxM1 overexpression led to a worse prognosis in CRC patients. Besides, overexpression of both Rab1A and FoxM1 led to a worse prognosis than AL group, and BL expression of Rab1A and FoxM1 had a better prognosis than AL group. Thus, Rab1A and FoxM1 play crucial roles and could be used as clinical biomarkers of CRC.

## Methods

### Patients and tissue specimens

The Rab1A and FOXM1 expression in gastrointestinal cancers was obtained via the GEPIA Platform (https://gepia.cancer-pku.cn), an online analysis website for The Cancer Genome Atlas (TCGA) and Gene Tissue Expression (GTEX) databases. The expression of Rab1A and FOXM1 was obtained from the Oncomine platform (https://www.oncomine.org/resource/login.html). Data extraction and analysis were performed according to the instructions for authors stated on the website. CRC tissues were collected from 135 patients from the Department of General Surgery, Suzhou Municipal Hospital (Suzhou, China) from 2010–2013. None of these patients had received radiotherapy or chemotherapy before radical surgery. All tissue specimens were verified using histopathological studies. Clinical data were obtained from hospital records and all specimens were obtained after permission. The study was supported by the Independent Ethics Committee (IEC) of Suzhou Municipal Hospital and all patients provided written informed consent. After surgery, each patient was followed-up regularly, as described in our previous research^19^.

### Immunohistochemistry (IHC)

IHC was performed to investigate the expression of Rab1A and FoxM1 in 135 CRC tissue samples and adjacent normal tissues. All analyses were performed in accordance with the relevant guidelines and regulations. Surgical specimens were fixed in 10% formalin, embedded in paraffin, cut into 5-μm sections, dewaxed, and then rehydrated. Next, the processed sections were blocked with 10% goat serum for 30 min and incubated with the polyclonal antibodies of Rab1A and FOXM1 at room temperature overnight. IHC was performed using a tissue staining kit (Zhongshan Biotechnology, Beijing, China) following the manufacturer’s protocol. Immunostaining was independently examined by two clinical pathologists who were blinded to the patient outcome. The staining score was calculated using Yang, K’s method^28^. The staining scores were as follows: 0 was deemed as (−),1–4 as (+), 5–8 as (++), and 9 –12 as (+++). In our current study, we classified all samples into a high expression group (++ or +++) or a low expression group (− or +) based on protein expression.

### Statistical analysis

Data are expressed as the means ± S.E.M. All statistical analyses were performed using SPSS22.0 software (SPSS Inc, Chicago, IL, USA), GraphPad Prism 8 (San Diego, CA) and R programs. Student’s *t*-test (unpaired, two-tailed) or one-way ANOVA were used to compare means between groups. Survival duration was calculated using the Kaplan–Meier method and compared using the log-rank test. IHC results were analyzed using Chi-squared or Fisher’s exact tests. Self-developed R program (version 3.6.2 for Windows, https://cran.r-project.org/) was used for Cluster analysis and Nomogram analysis. A value of *P* < 0.05 was considered to indicate a statistically significant difference.

### Consent for publication

All the individuals provided written informed consent prior to enrolling in the study, and the study was approved by the Ethics Committees of Suzhou Municipal Hospital.

## Data Availability

Data are stored by the corresponding author of this paper and are available upon request.
